# Case Report: Fabry cardiomyopathy associated with a novel truncating *GLA* variant, c.837_838delinsAT, p.(Gln280Ter)

**DOI:** 10.3389/fmed.2026.1970747

**Published:** 2026-09-28

**Authors:** Fernando Perretta, Eduardo Zurita, Marcelo Ortiz, Juan Politei

**Affiliations:** 1 Department of Nephrology, NEFRA Medical Care, Escobar, Argentina; 2 Intensive Care Unit, Hospital Zonal General de Agudos Dr. Enrique Erill, Escobar, Argentina; 3 Department of Cardiology, Hospital Zonal General de Agudos Dr. Enrique Erill, Escobar, Argentina; 4 Department of Neurology, Fundación SPINE, CABA, Argentina

**Keywords:** cardiac magnetic resonance, cardiogenomics, Fabry cardiomyopathy, Fabry disease, GLA gene, precision medicine

## Abstract

We report the comprehensive clinical characterization of a woman with Fabry disease (FD) carrying a previously unreported truncating *GLA* variant, c.837_838delinsAT, p.(Gln280Ter). The patient, a 52-year-old woman with longstanding hypertrophic cardiomyopathy, was referred to a specialized Fabry Disease Program to investigate FD as the underlying etiology of her cardiomyopathy. Cardiac magnetic resonance imaging demonstrated diffuse left ventricular hypertrophy with extensive late gadolinium enhancement, indicating advanced replacement fibrosis and an increased arrhythmic risk. Biochemical evaluation revealed reduced *α*-galactosidase A activity and markedly elevated Lyso-Gb3 levels. Comprehensive systemic assessment identified bilateral cornea verticillata and nonspecific periventricular white matter hyperintensities on brain magnetic resonance imaging, while renal function remained preserved without clinically significant renal involvement. Molecular analysis identified the heterozygous truncating *GLA* variant c.837_838delinsAT, p.(Gln280Ter), which had not previously been reported. The patient received an implantable cardioverter-defibrillator following cardiovascular risk assessment and subsequently initiated enzyme replacement therapy with agalsidase beta. This case expands the known mutational spectrum of the *GLA* gene and illustrates the value of integrating molecular genetic analysis with multimodality cardiac imaging and comprehensive phenotyping in patients with unexplained hypertrophic cardiomyopathy. This integrated approach not only supports the interpretation of previously unreported *GLA* variants but may also refine etiologic diagnosis, cardiovascular risk assessment, and individualized clinical management in FD.

## Introduction

Fabry disease (FD) is an inherited X-linked lysosomal disorder resulting from pathogenic variants in the *GLA* gene, which encodes the enzyme α-galactosidase A (α-Gal A). Reduced or absent α-Gal A activity leads to lysosomal accumulation of glycosphingolipids, predominantly globotriaosylceramide (Gb3) and its deacylated derivative globotriaosylsphingosine (Lyso-Gb3). Progressive substrate accumulation across multiple cell types results in a multisystem disorder primarily affecting the kidneys, heart, and nervous system. Although disease-specific therapies have significantly improved patient outcomes, their greatest benefit is achieved when treatment is initiated before irreversible organ damage has developed (1).

The molecular spectrum of FD is highly heterogeneous. To date, more than 2,200 *GLA* variants have been catalogued in ClinVar, encompassing pathogenic, likely pathogenic, benign, likely benign, and variants of uncertain significance (2). Consequently, the interpretation of previously unreported variants remains a significant diagnostic challenge. In these situations, assessment of pathogenicity relies not only on genetic findings but also on the integration of biochemical data, clinical manifestations, family history, and established variant interpretation criteria (3).

Cardiac involvement is among the most important determinants of prognosis in FD and may dominate the clinical presentation, particularly in heterozygous females. Disease severity ranges from asymptomatic or minimally symptomatic to severely affected phenotypes depending on the *GLA* variant, residual *α*-Gal A activity, and X-chromosome inactivation pattern. Left ventricular hypertrophy (LVH), replacement fibrosis detected by late gadolinium enhancement (LGE) on cardiac magnetic resonance (CMR), conduction abnormalities, and ventricular arrhythmias represent the principal cardiac manifestations and are associated with an increased risk of adverse cardiovascular events (4, 5).

We report the clinical characterization of a woman with FD in whom a novel truncating *GLA* variant was identified. The integration of genetic, biochemical, neurological, ophthalmological, and cardiac imaging findings highlights the value of detailed phenotyping for the interpretation of previously unreported *GLA* variants.

## Case description

A 52-year-old woman with a longstanding history of hypertrophic cardiomyopathy and supraventricular arrhythmia, who reported fatigue and palpitations and was receiving bisoprolol 2.5 mg once daily, was under cardiological follow-up. She had no known family history of cardiomyopathy, sudden cardiac death, FD, or kidney disease. Electrocardiography demonstrated sinus rhythm with electrocardiographic features of LVH, including increased QRS voltage and secondary repolarization abnormalities (Figure 1). Transthoracic echocardiography revealed moderate-to-severe concentric LVH with predominant apical involvement (maximum wall thickness, 17 mm), preserved left ventricular systolic function (Simpson’s ejection fraction, 66%), mild left atrial enlargement, and grade II diastolic dysfunction, without left ventricular outflow tract obstruction. Twenty-four-hour Holter monitoring showed sinus rhythm throughout the recording, with a low mean heart rate (48 bpm), frequent supraventricular premature beats, and a brief episode of atrial tachycardia, without ventricular arrhythmias or significant pauses. CMR confirmed diffuse LVH, most prominent at the apex (maximum wall thickness, 18 mm), with systolic apical cavity obliteration. Extensive LGE was observed, predominantly affecting the apicoanterior segments, consistent with extensive replacement myocardial fibrosis and associated with an increased arrhythmic risk (Figure 2).

**Figure 1:**
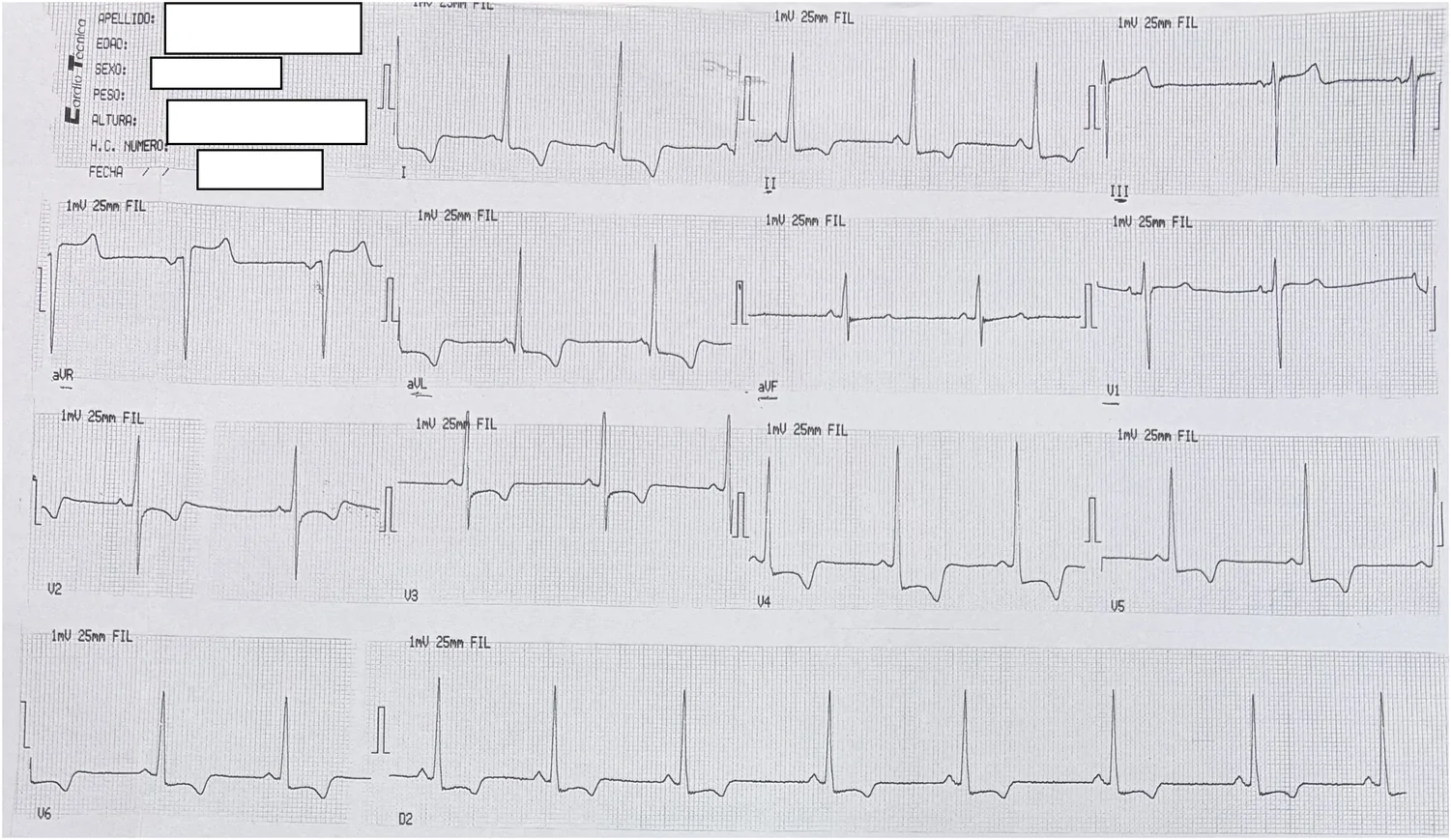
ECG with sinus rhythm and evidence of LVH, characterized by increased QRS voltage and secondary repolarization abnormalities (T wave inversions in precordial leads). ECG, electrocardiogram; LVH, left ventricular hypertrophy.

**Figure 2:**
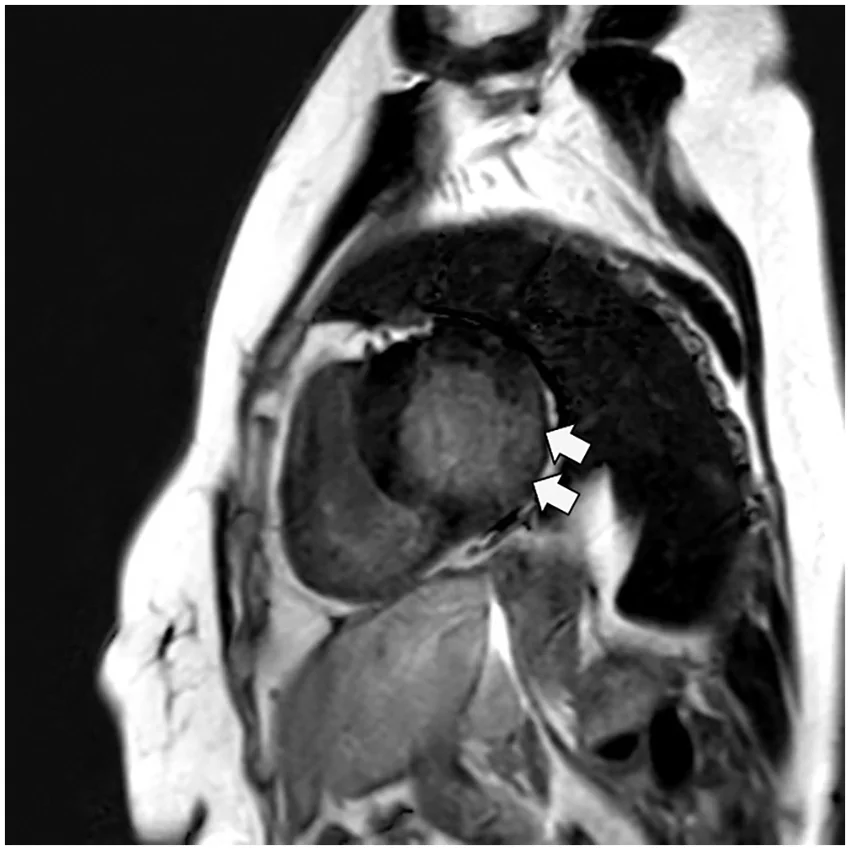
Representative short-axis CMR image showing LGE consistent with replacement myocardial fibrosis (arrows). CMR, cardiac magnetic resonance; LGE, late gadolinium enhancement.

## Diagnostic assessment

Based on the marked LVH and extensive myocardial fibrosis, the patient was referred by the Cardiology Department to the Fabry Disease Program of the Department of Nephrology at our institution in June 2024 to investigate whether FD could explain her hypertrophic cardiomyopathy. Because *α*-Gal A activity can remain within the normal range in heterozygous females due to random X-chromosome inactivation, Lyso-Gb3 was concurrently assessed. Dried blood spot analysis demonstrated reduced *α*-Gal A activity (1.3 μmol/L/h; reference >2.8 μmol/L/h) together with markedly elevated Lyso-Gb3 levels (12.9 ng/mL; reference 0.0–3.5 ng/mL). Molecular analysis of the *GLA* gene identified the heterozygous novel truncating variant c.837_838delinsAT, p.(Gln280Ter), initially classified as likely pathogenic. This classification was supported by the following ACMG/AMP criteria (3): PVS1 (null variant predicted to trigger nonsense-mediated mRNA decay in *GLA*, a gene in which loss-of-function is an established disease mechanism) and PM2 (absent from population databases, including gnomAD). The highly specific Fabry phenotype, including reduced *α*-Gal A activity, elevated Lyso-Gb3, bilateral cornea verticillata, and characteristic Fabry cardiomyopathy, provided additional phenotypic support for pathogenicity (PP4). A subsequent hypertrophic cardiomyopathy gene panel confirmed the same *GLA* premature termination codon variant and additionally identified a variant of uncertain significance in the transthyretin gene (*TTR*; p.Thr23Met). No clinical, biochemical, or imaging evidence of TTR-related amyloidosis was present, and this variant was therefore not considered to contribute to the patient’s cardiac phenotype.

Comprehensive systemic evaluation demonstrated bilateral cornea verticillata on slit-lamp examination. Brain magnetic resonance imaging revealed multiple nonspecific periventricular white matter hyperintensities without evidence of prior cerebrovascular events. Renal involvement was absent, with preserved renal function (serum creatinine 0.7 mg/dL), normal urinary albumin-to-creatinine ratio (12.8 mg/g), no clinically significant proteinuria, and normal renal ultrasonography.

## Therapeutic intervention, follow-up and outcomes

An implantable cardioverter-defibrillator (ICD) was placed in December 2024 following cardiovascular risk assessment, considering the marked LVH and extensive myocardial fibrosis on CMR. Given the presence of established cardiac involvement, and in accordance with consensus recommendations for FD management (1), enzyme replacement therapy (ERT) with agalsidase beta (Fabrazyme, Sanofi; 1 mg/kg every other week) was initiated in March 2025. Treatment has been well tolerated, with no infusion-associated reactions or treatment-related adverse events. The patient has remained clinically stable during follow-up, without new Fabry-related events, and continues under regular multidisciplinary care. The timeline of the patient’s clinical course is summarized in Figure 3.

**Figure 3:**
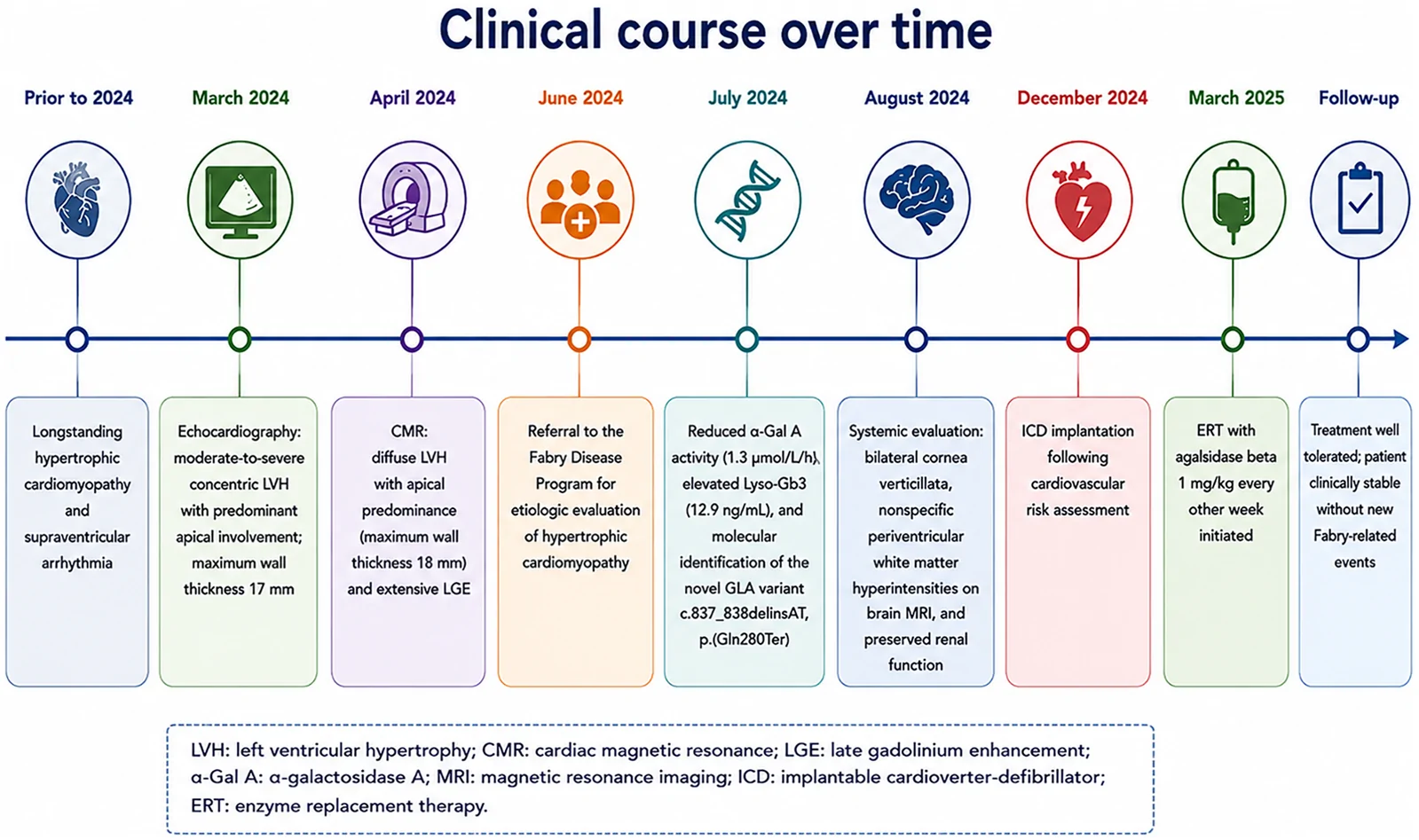
Clinical course over time.

## Discussion

This report describes the clinical characterization of a woman with FD carrying a previously unreported truncating *GLA* variant, p.(Gln280Ter). The diagnosis was established through the integration of clinical manifestations, reduced *α*-Gal A activity, elevated Lyso-Gb3 levels, multimodality cardiac imaging, and molecular testing. This case highlights the importance of integrating biochemical, imaging, and genetic findings when evaluating previously unreported *GLA* variants, particularly in women, in whom the diagnosis may be challenging because of the marked phenotypic variability of the disease (1, 5, 6).

Although FD is inherited as an X-linked disorder, heterozygous women should not be considered asymptomatic carriers. Clinical manifestations range from the absence of detectable organ involvement to severe multisystem disease, reflecting the combined influence of residual *α*-Gal A activity, X-chromosome inactivation, and other genetic and environmental modifiers. Consequently, *α*-Gal A activity may be normal or only mildly reduced in affected women, making molecular testing essential for diagnosis. In our patient, the coexistence of reduced *α*-Gal A activity, markedly elevated Lyso-Gb3 levels, bilateral cornea verticillata, and characteristic Fabry cardiomyopathy provided compelling evidence supporting the diagnosis of FD. Brain magnetic resonance imaging additionally demonstrated nonspecific periventricular white matter hyperintensities, a finding that has been described in patients with FD but is not disease specific (4–6).

Cardiac involvement is the leading cause of morbidity and mortality in FD and may be the predominant or even the presenting manifestation in women. Progressive LVH, myocardial fibrosis, conduction abnormalities, atrial and ventricular arrhythmias, and heart failure constitute the major features of Fabry cardiomyopathy. Because the cardiac phenotype may closely resemble sarcomeric hypertrophic cardiomyopathy, current recommendations emphasize the importance of considering FD in patients presenting with otherwise unexplained LVH (1, 5–7). In the present case, referral from the Cardiology Department to a dedicated Fabry Disease Program was crucial for establishing the correct etiologic diagnosis and enabling timely initiation of ERT.

CMR played a pivotal role in defining disease severity. The presence of LGE is a well-established marker of replacement myocardial fibrosis and advanced Fabry cardiomyopathy (5, 8). Although LGE most commonly involves the basal inferolateral wall, more extensive or atypical distributions have been described in advanced disease (8, 9). In the present case, CMR demonstrated extensive LGE, predominantly affecting the apicoanterior segments, consistent with a substantial burden of replacement myocardial fibrosis. Importantly, Hanneman et al. demonstrated that patients with extensive LGE (≥15% of LVM) have a markedly increased risk of adverse cardiac outcomes, including ventricular arrhythmias, heart failure progression, and cardiovascular death (10). These findings reinforce the pivotal role of CMR not only for diagnosis but also for risk stratification and therapeutic decision-making in FD (7–10).

Although the p.(Gln280Ter) variant has not previously been reported in ClinVar or other major variant databases, its predicted molecular consequence is consistent with the established loss-of-function mechanism of *GLA* pathogenic variants (2, 3). More importantly, the molecular finding was fully concordant with the patient’s previously described phenotype. This comprehensive phenotypic characterization, integrating molecular genetic, biochemical, clinical, and cardiac imaging findings, was essential to support the pathogenicity of this novel variant and illustrates the broader value of detailed genotype-phenotype correlation in the interpretation of previously unreported *GLA* variants encountered in clinical practice. Such an integrated approach is particularly relevant to precision medicine, in which molecular findings are interpreted within the individual patient’s phenotypic and imaging context to inform diagnosis, risk stratification, and clinical management (1, 6, 11, 12).

A major strength of this case is the comprehensive multidisciplinary characterization integrating molecular genetic, biochemical, clinical, and cardiac imaging findings. However, as a single-patient case report, the genotype–phenotype association cannot be generalized, and longer-term follow-up will be required to assess clinical outcomes.

Finally, this case emphasizes the importance of considering FD in women presenting with unexplained hypertrophic cardiomyopathy, particularly when CMR demonstrates extensive myocardial fibrosis or atypical patterns of LGE. The integration of molecular genetic testing, biomarkers, comprehensive phenotyping, and advanced cardiac imaging enabled etiologic diagnosis, cardiovascular risk assessment, and disease-specific therapeutic management in this patient. This case expands the genotype–phenotype spectrum of FD and illustrates how an integrated precision-medicine approach may facilitate the interpretation of novel *GLA* variants and support individualized cardiovascular care.

## Patient perspective

The patient described the prolonged uncertainty regarding the cause of her cardiac disease, together with multiple medical consultations and repeated diagnostic studies, as a distressing experience. Identifying FD as the underlying cause of her cardiomyopathy was perceived as a relief, particularly because a disease-specific treatment was available.

## Data Availability

The original contributions presented in the study are included in the article; further inquiries can be directed to the corresponding author.
